# Nonisothermal Crystallization Kinetics of Acetylated Bamboo Fiber-Reinforced Polypropylene Composites

**DOI:** 10.3390/polym11061078

**Published:** 2019-06-22

**Authors:** Yu-Shan Jhu, Teng-Chun Yang, Ke-Chang Hung, Jin-Wei Xu, Tung-Lin Wu, Jyh-Horng Wu

**Affiliations:** 1 Department of Forestry, National Chung Hsing University, Taichung 402, Taiwan; j1129w@gmail.com (Y.-S.J.); tcyang.04@nchu.edu.tw (T.-C.Y.); d9833004@mail.nchu.edu.tw (K.-C.H.); ecsgunro@gmail.com (J.-W.X.); 2 College of Technology and Master of Science in Computer Science, University of North America, Fairfax, VA 22033, USA; tonywuwu22@gmail.com

**Keywords:** acetylation, bamboo fiber, cooling rate, crystallization kinetics, nonisothermal crystallization, polypropylene (PP)

## Abstract

The crystallization behavior of bamboo fiber (BF) reinforced polypropylene (PP) composites (BPCs) was investigated using a differential scanning calorimeter (DSC). The results showed that unmodified BF as a nucleation agent accelerated the crystallization rate of the PP matrix during cooling whereas there is no significant effect on the improved crystallization rate in BPCs with acetylated BFs. Based on the Avrami method, Avrami–Ozawa method, and Friedman method, the corresponding crystallization kinetics of PP reinforced with different acetylation levels of BFs were further analyzed. The results demonstrated that the crystal growth mechanism of the PP matrix for BPCs with unmodified and various acetylated BFs exhibited tabular crystal growth with heterogeneous nucleation. A higher cooling rate is required to achieve a certain relative crystallinity degree at the unit crystallization time for BPCs with a higher weight percent gain (WPG) of acetylated BFs (WPG >13%). Furthermore, based on the Friedman method, the lowest crystallization activation energy was observed for the BPCs with 19% WPG of acetylated BFs.

## 1. Introduction

Natural fibers have potential as an eco-friendly reinforcement of composite materials to replace inorganic fibers due to their CO_2_ neutral, low cost, low density, renewability, and biodegradability properties [1]. Recently, wood plastic composites (WPCs), which are thermoplastics reinforced with natural fibers, are of growing interest in various applications such as automotive and building construction. Among natural fibers, bamboo fiber (BF) has a faster growth rate than other plants composed of fibers and is widely available across Asia [2]. Additionally, previous studies have reported that BFs as a reinforcement can be added to a polymer matrix to fabricate bamboo plastic composites (BPCs) since their excellent characteristics could improve the properties of these polymer composites [3,4,5,6,7,8,9,10]. However, it is well-known that the interfacial interaction between hydrophilic lignocellulosics and hydrophobic thermoplastics is incompatible. Thus, to overcome this drawback, several physical and chemical approaches have been used to modify lignocellulosic fibers, e.g., by increasing their hydrophobicity and improving their dimensional and thermal stabilities [11,12,13,14]. Among these various approaches, acetylation as an important chemical method for the modification of lignocellulosic fibers has received the most attention. Many studies have reported that this method improved the compatibility between fiber/polymer interfaces. Lisperguer et al. [15] reported that WPCs reinforced with acetylated wood fibers (WFs) exhibit higher thermal stability than those reinforced with untreated WFs. Hung et al. [16] investigated the influence of acetylated BFs with different weight percentage gains (WPGs) on the natural weathering properties of bamboo plastic composites. The results showed that the retention ratios of the mechanical properties of composites with a high WPG were significantly improved during natural weathering. Hung et al. [17] also studied the effect of wood acetylation on the mechanical properties and creep resistance of WPCs. The result showed the WPCs with acetylated WFs have superior mechanical properties, mildew resistance, and creep resistance compared to that of WPCs with unmodified WFs, especially for WPC with 13% WPG acetylated WFs. To date, studies have mainly focused on the effect of acetylated WFs on the thermal and mechanical properties of WPCs. However, the resulting microstructure of the polymer matrix, as reflected by crystallization and orientation, has a significant effect on the quality and properties of the end products since it is dependent on the press and thermal history during the manufacturing process [18]. In addition, the composites are solidified under nonisothermal conditions in extrusion and compression moldings. Therefore, the crystallization mechanism of the final composite material is influenced by the nonisothermal crystallization behavior of the WPCs. Many studies have investigated the crystallization kinetics of polymer composites with various natural fibers [19,20,21,22,23,24]. However, to the best of our knowledge, the effect of acetylated bamboo fibers on the nonisothermal crystallization behavior of BF/polymer composites has not been clearly indicated. In this study, the crystallization behavior of BPCs with acetylated BFs under nonisothermal conditions was examined using differential scanning calorimetry (DSC) analysis. In addition, the Avrami method and Avrami–Ozawa method were used to investigate the nucleation mechanism and the required crystallization rate, respectively. Finally, the activation energy of nonisothermal crystallization was analyzed using the Friedman method.

## 2. Experimental Section

### 2.1. Materials

PP pellets were purchased from Yung Chia Chemical Industries Co., Ltd. (Taipei, Taiwan). Its density, melt flow index, and melting point were 915 kg/m^3^, 4–8 g/10 min, and 165 °C, respectively. These plastic pellets were ground in an attrition mill to reduce their particle size to less than 100 mesh (<150 μm). Bamboo fibers (BFs) were prepared from 3-year-old kei-chiku bamboo (*Phyllostachys makinoi* Hayata) shavings, which were kindly provided by the local bamboo-processing factory, via hammer-milling and sieving to obtain fibers between 24 and 30 mesh (700‒550 μm). All BFs were used after extraction in a Soxhlet apparatus for 24 h with acetone, followed by washing with distilled water before drying at 75 °C for 24 h. Acetic anhydride (AA), dimethylformamide (DMF), and acetone were purchased from Sigma-Aldrich Chemical Co. (St. Louis, MO, USA).

### 2.2. Bamboo Acetylation

BFs were acetylated with AA using the liquid-phase reaction method. The concentration ratios of AA and DMF were 0.2/20, 0.3/20, 1/20, 2/20, and 4/20 (mL/mL) per gram of oven-dried BF. The reaction was conducted at 140 °C for 2 h to obtain acetylated BFs with different degrees of modification. At the end of the reaction, the acetylated fibers were washed with distilled water for 4 h and dried at 105 °C for 12 h, and the weight percent gain (WPG) of the acetylated fibers was calculated from Equation (1).

(1) $$\mathrm{WPG}\;\left(\%\right)=\left[\left(w_{1}-w_{0}\right)/w_{0}\right]\times 100$$

where *w*_0_ and *w*_1_ are the weight of an oven-dried sample before and after immersion, respectively. The resulting WPGs of acetylated BFs were approximately 2, 6, 9, 13, and 19% in the present study. For composites, the weight ratio of the acetylated BFs to PP powder was 50/50. Additionally, the sample codes of PP with unmodified and various acetylated BFs are shown in Table 1.

### 2.3. Differential Scanning Calorimetry (DSC)

A total of 3–5 mg of each sample was prepared directly in DSC aluminum pans with lids. The nonisothermal crystallization was executed in nitrogen at a flow rate of 20 mL/min using a PerkinElmer DSC-6 (Beaconsfield, UK). To ensure a nuclei-free melt and eliminate the thermal and mechanical history, the first step was preliminary heating to 200 °C with a heating rate of 10 °C/min, and this temperature was held for 5 min. The samples were cooled to 50 °C at different constant cooling rates of 2.5, 5, 10, 15, 20, and 25 °C/min. The exothermic flow curves were recorded to analyze the nonisothermal crystallization kinetics. Three individual samples were tested for each composition. The PP crystallinity (*X*_c_) was obtained as a function of the cooling rate using the following expression (Equation (2)):

(2) $$X_{c}\left(\%\right)=\frac{\Delta H_{m}}{\Delta H_{m}^{0}\times W_{p}}$$

where Δ*H*_m_ is the melting enthalpy for the sample at a given cooling rate, $\Delta H_{m}^{0}$ denotes the melting enthalpy of fully crystalline PP, which was 209 J/g [25], and *W*_p_ denotes the weight percentage of PP in the samples.

### 2.4. Nonisothermal Crystallization Kinetics

The relative crystallinity (*X*) as a function of crystallization temperature (*T*) can be formulated based on the nonisothermal DSC data using Equation (3).

(3) $$X\left(\%\right)=\int_{T_{0}}^{T}\left(\frac{dH_{c}}{dT}\right)dT/\int_{T_{0}}^{T_{\infty }}\left(\frac{dH_{c}}{dT}\right)dT$$

where *dH*_c_/*dT* is the heat flow rate and *T*_0_ and *T*_∞_ are the onset and offset temperatures of crystallization, respectively. The crystallization time (*t*) at a given cooling rate can be calculated from Equation (4).

(4) $$t=\left(T_{0}-T\right)/\phi$$

where *T*_0_ is the onset temperature at crystallization time *t* = 0, *T* is the temperature at time *t*, and *φ* is the cooling rate.

Based on the Avrami (Equation (5)) [26,27,28] and Ozawa (Equation (6)) approaches [29], the crystallization kinetics were determined using the following equations:

(5) $$X\left(t\right)=1-e^{-Kt^{n}}$$

(6) $$-\ln\left(1-X\left(T\right)\right)=\frac{k\left(T\right)}{\phi^{m}}$$

where *X*(*t*) is the relative crystallinity at time *t*, *K* is the Avrami crystallization rate constant, *n* denotes a mechanism constant, *X*(*T*) is the relative crystallinity at temperature *T*, *k*(*T*) is the cooling crystallization function, *m* is the Ozawa exponent, and *φ* is the constant cooling rate. Furthermore, Equations (5) and (6) can be converted to Equations (7) and (8) by taking the logarithm of both sides, respectively.

(7) log[−ln(1−X(t))]=nlog t+log K

(8) log[−ln(1−X(T))]=log k(T)−mlog ϕ

However, previous studies have reported that the Ozawa equation may not be appropriate for describing the nonisothermal crystallization kinetics of some polymer systems [30,31,32]. Therefore, an equation related to the cooling crystallization rate (*φ*) and time (*t*) could be generated at a given degree of crystallization by combining the Avrami and Ozawa equations [33,34] as the following Equation (9):

(9) ln ϕ=ln F(T)−αln t

where *F*(*T*) = [*k*(*T*)/*K*]^1/*m*^ denotes the value of the cooling rate at the unit crystallization time under a given degree of crystallinity and *α* denotes the ratio of the Avrami exponent (*n*) to the Ozawa exponent (*m*).

Moreover, the crystallization activation energy was calculated by the Friedman equation (Equation (10)), and the equation is expressed as follows [35]:

(10) $$\ln{\left(\frac{dX}{dt}\right)}_{X}=\ln\left[A_{X}f\left(X\right)\right]-\frac{E_{X}}{RT_{X}}$$

where *X* is the relative degree of crystallinity, *t* is the time, *f*(*X*) is the function of the reaction mechanism, *R* is the universal gas constant, and *A_X_*, *E_X_*, and *T_X_* are the pre-exponential factor, activation energy, and temperature at a given *X* value, respectively.

### 2.5. Statistical Analysis

All results are expressed as the mean ± standard deviation (SD). The significance of the differences was calculated by Scheffe’s test, and *p* values < 0.05 were considered significant.

## 3. Results and Discussion

### 3.1. Nonisothermal Crystallization Behavior

**Figure 1:**
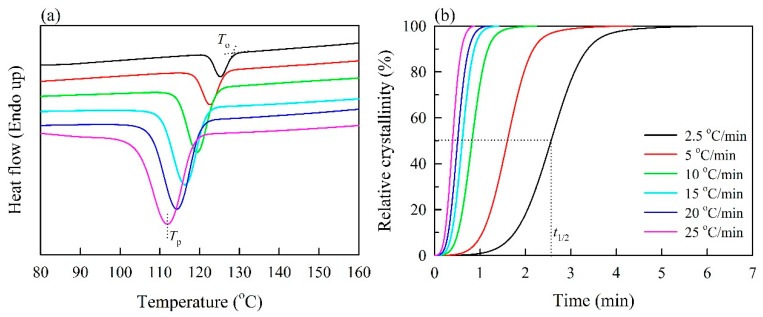
Nonisothermal crystallization behaviors of polypropylene with unmodified bamboo fibers. (**a**) Differential scanning calorimetry (DSC) thermograms. (**b**) Relative crystallinity as a function of time.

DSC thermogram curves for the nonisothermal crystallization of PP with unmodified BFs (BPC_0_) are presented for a range of cooling rates from 2.5 to 25 °C /min in Figure 1a. The onset temperature (*T*_o_) and crystallization peak (*T*_p_) shifted to lower temperatures for all samples with an increasing cooling rate. For example, as shown in Table 2, the *T*_o_ of BPC_0_ significantly decreased from 127.2 °C at 2.5 °C/min to 117.3 °C at 25 °C/min. At lower cooling rates, the initiation of crystallization occurred at higher temperatures due to sufficient time to induce nuclei. In contrast, crystallization occurred at lower temperatures because the motion of PP chains fell behind the higher cooling rate [22,36]. Additionally, the effects of acetylated BFs on the *T*_o_ and *T*_p_ of BPCs were investigated. At a given cooling rate, the values of BPCs decreased with increasing WPG of acetylated BFs. At 25 °C/min, the *T*_o_ and *T*_p_ significantly decreased from 117.3 °C and 111.0 °C (BPC_0_) to 112.5 °C and 107.3 °C when the WPG of acetylated BFs reached 19%. However, there were no significant differences in crystallinity (*X*_c_) among all samples in the range of 45% to 70%, indicating that these values were independent of the WPG of acetylated BFs. Additionally, as shown in Figure 1b, the relative crystallinity (*X*) as a function of crystallization time (*t*) for BPC_0_ at various cooling rates can be formulated according to Equations (3) and (4). All curves of BPC_0_ at different cooling rates showed sigmoidal growth, and nearly all BPCs exhibited the same trend. Previous studies [30,33,37] have reported that the trend of these curves was attributed to a lag effect of the cooling rate on crystallization, resulting in two crystallization processes: A fast crystallization process (the initial stage) and a slower crystallization process (the later stage). At the later stage of the curve, the crystallization rate obviously decreased due to the impingement of spherulites [38]. Additionally, compared to the higher cooling rate, the curve ramp was lower at the slower cooling rate. This result indicated that the increase in cooling rates accelerated the crystallization completion of the PP matrix [23,24]. This phenomenon was further confirmed by the half-life of crystallization (*t*_1/2_), which is defined as the period corresponding to 50% of *X*. As shown in Table 2, the *t*_1/2_ values of all samples fell with increasing cooling rates. For example, the *t*_1/2_ value of the BPC_0_ significantly decreased from 2.31 min at 2.5 °C/min to 0.41 min at 25 °C/min. Furthermore, the *t*_1/2_ value of BPC_0_ was lower than that of BPCs with acetylated BFs, and the effect of acetylated BFs was clear in the case of lower cooling rates. At 2.5 °C/min, the *t*_1/2_ value increased from 2.31 min to 2.81 min as the WPG of acetylated BFs increased to 2%. Once the WPG reached 19%, however, a significant increase in the *t*_1/2_ value was observed (ca. 3.17 min). Apparently, unmodified BFs acted as a heterogeneous nucleation agent to accelerate the crystallization of the PP matrix. A similar result was reported in our previous study [23]. However, the acetylated BFs decreased the crystallization rate of the PP matrix with increasing WPG. This result may be attributed to the fact that acetylated BFs with a higher WPG (i.e., more hydrophobic acetyl moieties attached to the fiber surface) caused homogeneous crystal growth.

### 3.2. Avrami Model

**Figure 2:**
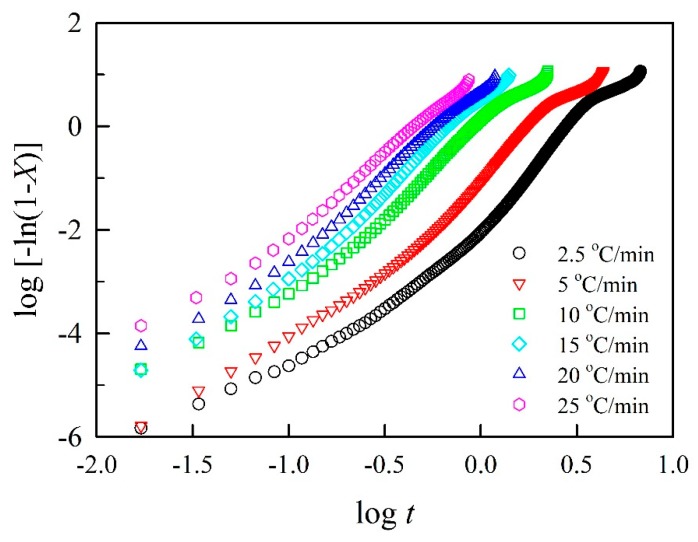
Avrami plots of log[−ln(1−*X*)] versus log *t* for polypropylene with unmodified bamboo fibers.

The nucleation mechanisms of BPCs were analyzed using the Avrami approach. The Avrami model is based on the isothermal crystallization kinetics and has been widely used to describe the initial stage of crystallization for polymers under the assumption that the crystallization temperature is constant. According to Equation (5), the values of *n* and *K* from the slope of the linear portion and intercept of the curve can be obtained from a plot of log[−ln(1 − *X*(*t*))] versus log *t*. As shown in Figure 2, linear fits of Avrami plots were conducted for log[−ln(1 − *X*(*t*))] values from –0.45 to –3.50, and the corresponding crystallinity is 0% to 30%. In Table 3, the square of the correlation coefficient (*R*^2^) value of each linear regression on the slope was greater than 0.95 for all samples. However, the *n* values of BPC_0_ were 2.72–3.00 at different cooling rates and ranged from 2.57 to 3.09 for BPCs with various acetylated BFs. Additionally, the *n* values had no significant differences among the samples, suggesting that the nonisothermal crystallization mechanisms of PP were tabular crystal growth with heterogeneous nucleation. These results indicated that the nucleation mechanism and geometry of crystal growth at different cooling rates were similar; the acetylated BF was not significantly different from the unmodified BF in the nucleation mechanism of the PP matrix under the cooling process. Moreover, the nonisothermal crystallization rate (*K*_J_) was calculated using the following equation [39]: log *K*_J_ = log *K*/*φ*. As shown in Table 3, the *K*_J_ values for all samples were markedly increased with increasing cooling rates up to 10 °C/min, indicating that the highest cold crystallization rate occurred at 10 °C/min. Compared to BPCs with unmodified and acetylated BFs, the *K*_J_ values exhibited no significant differences among all BPCs at the given cooling rate. This result indicated that the WPG of acetylated BF was independent of the crystallization rate of the PP matrix.

### 3.3. Avrami–Ozawa Model

**Figure 3:**
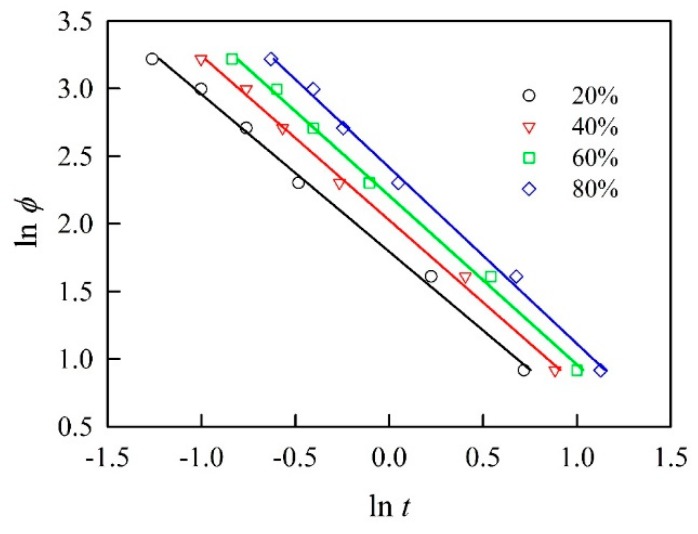
Avrami–Ozawa plots of ln *φ* versus ln *t* for polypropylene with unmodified bamboo fibers.

To further validate the accuracy of the crystallization behavior, the overall nonisothermal crystallization kinetics of BPCs were elaborated with the Avrami–Ozawa approach. As shown in Figure 3, the characteristic plot of ln *φ* versus ln *t* of BPC_0_ at various relative crystallinities is presented according to Equation (9). Each crystallization stage was fitted with a straight line, and the *R*^2^ value was greater than 0.97. This result demonstrated that this model is applicable for describing the nonisothermal crystallization kinetics of the BPCs with unmodified and acetylated BFs. At a given relative crystallinity, *F*(*T*) and *α* can be obtained from the intercept and the slope of a series of straight lines in the Avrami–Ozawa plot, respectively. As shown in Table 4, the *F*(*T*) of all samples increased with increasing *X*, but the values of *α* were almost constant. Furthermore, at a 20% relative crystallinity, the *F*(*T*) values of BPC_19_ significantly increased from 5.7 for BPC_0_ to 6.6, which means that a higher cooling rate is required to achieve a given degree of crystallinity for BPCs with a higher WPG (~19%) of acetylated BFs. The *α* value remained constant for all BPCs with acetylated BFs at 20% relative crystallinity, while the value significantly decreased with increasing WPGs of BFs when the relative crystallinity was above 20%. This result confirmed a slow crystallization rate for BPCs with a higher WPG of acetylated BFs at higher relative crystallinity.

### 3.4. Friedman Model

**Figure 4:**
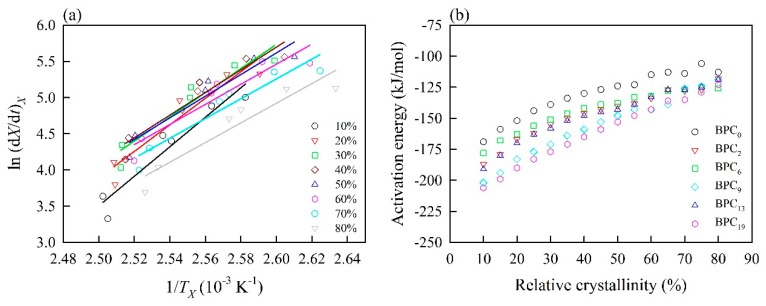
Nonisothermal crystallization kinetics of PP with unmodified and acetylated bamboo fibers using the Friedman method. (**a**) Friedman plots of ln (d*X*/d*t*)*_X_* versus 1/*T_X_* for PP with unmodified bamboo fibers; (**b**) dependence of the effective activation energy on the relative crystallinities.

In composites, two factors affect the crystallization of a polymer matrix. One corresponds to the free barrier energy of nucleation; the other is related to the activation energy for the transport of the macromolecular segments to the surface of crystal growth [39]. Therefore, crystallization activation energy is an indispensable parameter in polymer systems. Among the various kinetic models, the isoconventional Friedman model was used to estimate the crystallization activation energy in this study, since it is considered for nonisothermal crystallization as a function of relative crystallinity. Figure 4a illustrates the ln (*dX*/*dt*)*_X_* versus 1/*T_X_* plot for BPC_0_ at different relative crystallinities. Based on Equation (10), the activation energies (*E_X_*) for the crystallization process at a given relative crystallinity can be obtained from the slope of the straight line of the Friedman plots for various BPCs. As shown in Figure 4b, the *E_X_* values of all BPCs increased with increasing *X* from 5 to 80%. The resulting activation energies ranged from −169 to −113 kJ/mol, from −187 to −118 kJ/mol, from −178 to −126 kJ/mol, from −202 to −119 kJ/mol, from −191 to −119 kJ/mol, and from −206 to −123 kJ/mol for BPC_0_, BPC_2_, BPC_6_, BPC_9_, BPC_13_, and BPC_19_, respectively. These results suggested that the crystallization process was facile at the initial stage of crystallization but became more difficult when higher crystallinity was achieved. Additionally, in comparison with BPC_0_, a significant decrease in the *E_X_* value for BPCs with various acetylated BFs was observed. Among them, BPC_19_ exhibited the lowest energy barrier at the given *X*, except when *X* exceeded 60%. According to previous studies [23,24], the polymer composite with a lower energy barrier showed a higher crystallization rate of the polymeric matrix. However, the lowest crystallization rate of the BPC_19_ was observed by analyzing the DSC thermogram in the present study (Table 2). This phenomenon can be explained as the crystal growth types of the PP matrix (e.g., transcrystallization or spherulitic growth) are different for BPCs with various WPG of acetylated BFs. In the future, the aim of our study is to further investigate the effect of acetylated BFs on the crystal growth types of PP matrix in BPCs.

## 4. Conclusions

The nonisothermal crystallization kinetics of BPCs with various WPGs of acetylated BFs were determined by DSC and analyzed using the Avrami method, Avrami–Ozawa method, and Friedman method. The Avrami–Ozawa method showed that a higher cooling rate is required to obtain a certain relative crystallinity degree of the PP matrix for BPCs with higher WPG (~19%) of acetylated BFs within the unit crystallization time. Furthermore, the iso-conversional Friedman method showed that BPCs with 19% WPG of acetylated BFs exhibited the lowest energy barrier among all samples. According to the Avrami method, the result indicated that the unmodified BF addition could accelerate the crystal growth of the polymer matrix, while the addition of various WPGs of acetylated BFs would not significantly improve the crystallization rate of the polymer chains. From these methods, the cooling rate of unmodified and acetylated BFs significantly affected the crystallization mechanism, crystallization rate, and activation energy for the PP matrix. Accordingly, the results confirmed that the improved properties of BPCs with acetylated BFs are caused by factors such as transcrystallization of the PP matrix, compatibility of the BF/PP interface, and the properties of acetylated BFs, rather than the crystallization rate, relative crystallinity, and crystallization mechanism. To optimize the polymer morphology in a composite with acetylated BFs, these results offer information regarding manufacturing conditions.

## Figures and Tables

**Table 1:** Sample code and reaction conditions for different extents of acetylated BFs.

BPCs	BF:AA (g:mL)	Reaction Time (min)	WPG of Acetylated BF (%)	Acetylated BF/PP (wt%)
BPC_0_	-	-	0	50/50
BPC_2_	1:0.2	120	2.3 ± 0.9	50/50
BPC_6_	1:0.3	120	5.9 ± 1.1	50/50
BPC_9_	1:1	120	8.7 ± 1.6	50/50
BPC_13_	1:2	120	12.7 ± 0.8	50/50
BPC_19_	1:4	120	19.0 ± 1.5	50/50

Values are the mean ± SD (*n* = 5).

**Table 2:** Nonisothermal crystallization parameters as a function of cooling rate for polypropylene with unmodified and acetylated bamboo fibers.

Cooling Rate (°C/min)	BPCs	*T*_o_ (°C )	*T*_p_ (°C )	*X*_c_ (%)	*t*_1/2_ (min)
2.5	BPC_0_	127.2 ± 0.0 ^a^	124.3 ± 0.1 ^a^	67 ± 8 ^a^	2.31 ± 0.23 ^b^
	BPC_2_	127.2 ± 0.2 ^a^	123.1 ± 0.7 ^a^	56 ± 15 ^a^	2.81 ± 0.11 ^ab^
	BPC_6_	126.0 ± 0.2 ^a^	122.5 ± 0.1 ^a^	67 ± 5 ^a^	2.91 ± 0.11 ^ab^
	BPC_9_	125.8 ± 0.0 ^ab^	122.4 ± 0.1 ^ab^	64 ± 9 ^a^	2.71 ± 0.16 ^ab^
	BPC_13_	125.6 ± 0.3 ^ab^	122.5 ± 0.3 ^ab^	64 ± 4 ^a^	2.63 ± 0.16 ^ab^
	BPC_19_	124.3 ± 0.1 ^b^	120.8 ± 0.2 ^b^	60 ± 11 ^a^	3.17 ± 0.34 ^a^
5	BPC_0_	126.5 ± 1.7 ^a^	122.4 ± 1.4 ^a^	63 ± 9 ^a^	1.57 ± 0.10 ^ab^
	BPC_2_	125.9 ± 1.2 ^a^	121.7 ± 1.2 ^a^	53 ± 8 ^a^	1.61 ± 0.02 ^a^
	BPC_6_	124.6 ± 1.1 ^a^	120.3 ± 1.0 ^a^	67 ± 3 ^a^	1.57 ± 0.04 ^a^
	BPC_9_	123.8 ± 0.6 ^ab^	119.9 ± 0.4 ^ab^	59 ± 9 ^a^	1.41 ± 0.02 ^b^
	BPC_13_	123.7 ± 0.8 ^ab^	120.0 ± 0.7 ^ab^	57 ± 2 ^a^	1.39 ± 0.01 ^b^
	BPC_19_	121.5 ± 0.2 ^b^	117.6 ± 0.2 ^b^	60 ± 5 ^a^	1.57 ± 0.05 ^ab^
10	BPC_0_	122.1 ± 0.5 ^a^	117.6 ± 0.7 ^a^	64 ± 10 ^a^	0.82 ± 0.01 ^a^
	BPC_2_	122.4 ± 0.4 ^a^	117.4 ± 0.4 ^a^	66 ± 9 ^a^	0.91 ± 0.01 ^a^
	BPC_6_	122.1 ± 0.8 ^a^	116.9 ± 0.7 ^a^	62 ± 8 ^a^	0.90 ± 0.02 ^a^
	BPC_9_	120.7 ± 0.9 ^a^	116.0 ± 0.7 ^a^	63 ± 6 ^a^	0.81 ± 0.06 ^a^
	BPC_13_	120.9 ± 0.3 ^a^	116.5 ± 0.4 ^a^	64 ± 9 ^a^	0.84 ± 0.09 ^a^
	BPC_19_	118.1 ± 0.2 ^b^	113.7 ± 0.3 ^b^	60 ± 5 ^a^	0.96 ± 0.04 ^a^
15	BPC_0_	121.9 ± 1.7 ^a^	116.2 ± 1.3 ^a^	70 ± 7 ^a^	0.65 ± 0.03 ^a^
	BPC_2_	120.3 ± 0.1 ^ab^	115.1 ± 0.2 ^ab^	45 ± 18 ^a^	0.58 ± 0.05 ^a^
	BPC_6_	119.5 ± 1.0 ^ab^	113.3 ± 0.8 ^bc^	57 ± 4 ^a^	0.66 ± 0.02 ^a^
	BPC_9_	118.4 ± 0.7 ^bc^	113.1 ± 0.8 ^bc^	59 ± 14 ^a^	0.63 ± 0.06 ^a^
	BPC_13_	118.3 ± 0.2 ^bc^	113.3 ± 0.1 ^bc^	57 ± 1 ^a^	0.58 ± 0.05 ^a^
	BPC_19_	115.8 ± 0.3 ^c^	111.1 ± 0.3 ^c^	49 ± 5 ^a^	0.60 ± 0.04 ^a^
20	BPC_0_	118.7 ± 0.5 ^a^	112.8 ± 0.9 ^a^	59 ± 3 ^a^	0.48 ± 0.04 ^a^
	BPC_2_	118.3 ± 0.4 ^ab^	111.9 ± 0.9 ^a^	60 ± 3 ^a^	0.53 ± 0.04 ^a^
	BPC_6_	118.1 ± 0.3 ^ab^	111.4 ± 0.2 ^ab^	61 ± 7 ^a^	0.53 ± 0.02 ^a^
	BPC_9_	116.7 ± 0.6 ^ab^	111.2 ± 0.6 ^ab^	56 ± 5 ^a^	0.47 ± 0.04 ^a^
	BPC_13_	116.6 ± 0.4 ^b^	111.0 ± 0.3 ^ab^	59 ± 12 ^a^	0.46 ± 0.01 ^a^
	BPC_19_	114.0 ± 0.3 ^c^	108.9 ± 0.8 ^b^	50 ± 14 ^a^	0.48 ± 0.03 ^a^
25	BPC_0_	117.3 ± 0.3 ^a^	111.0 ± 0.5 ^a^	69 ± 10 ^a^	0.41 ± 0.01 ^a^
	BPC_2_	117.5 ± 0.6 ^a^	110.8 ± 0.5 ^a^	56 ± 9 ^a^	0.42 ± 0.00 ^a^
	BPC_6_	116.6 ± 1.8 ^a^	109.9 ± 1.6 ^ab^	62 ± 6 ^a^	0.44 ± 0.03 ^a^
	BPC_9_	115.4 ± 0.2 ^a^	109.6 ± 0.2 ^a^	51 ± 18 ^a^	0.38 ± 0.05 ^a^
	BPC_13_	115.5 ± 0.2 ^a^	109.6 ± 0.3 ^a^	64 ± 1 ^a^	0.39 ± 0.01 ^a^
	BPC_19_	112.5 ± 0.3 ^b^	107.3 ± 0.3 ^b^	56 ± 1 ^a^	0.38 ± 0.01 ^a^

Values are the mean ± SD (*n* = 3). Different superscript letters (a, b, and c) within a given cooling rate indicate a significant difference (*p* < 0.05).

**Table 3:** Parameters calculated at various cooling rates based on the Avrami model.

Cooling Rate (°C/min)	BPCs	*n*	*K*	*K*_J_	*R* ^2^
2.5	BPC_0_	2.82 ± 0.06 ^a^	0.03 ± 0.01 ^a^	0.24 ± 0.04 ^a^	0.974
	BPC_2_	2.95 ± 0.07 ^a^	0.01 ± 0.00 ^a^	0.17 ± 0.02 ^a^	0.963
	BPC_6_	2.54 ± 0.16 ^a^	0.01 ± 0.01 ^a^	0.18 ± 0.01 ^a^	0.951
	BPC_9_	2.64 ± 0.07 ^a^	0.02 ± 0.00 ^a^	0.19 ± 0.02 ^a^	0.956
	BPC_13_	2.80 ± 0.11 ^a^	0.02 ± 0.00 ^a^	0.18 ± 0.02 ^a^	0.961
	BPC_19_	2.75 ± 0.08 ^a^	0.01 ± 0.00 ^a^	0.16 ± 0.03 ^a^	0.963
5	BPC_0_	3.00 ± 0.13 ^a^	0.07 ± 0.01 ^a^	0.59 ± 0.01 ^a^	0.974
	BPC_2_	2.57 ± 0.02 ^a^	0.15 ± 0.13 ^a^	0.65 ± 0.12 ^a^	0.970
	BPC_6_	2.81 ± 0.02 ^a^	0.09 ± 0.01 ^a^	0.62 ± 0.01 ^a^	0.976
	BPC_9_	2.85 ± 0.09 ^a^	0.11 ± 0.01 ^a^	0.64 ± 0.01 ^a^	0.974
	BPC_13_	2.90 ± 0.17 ^a^	0.11 ± 0.00 ^a^	0.64 ± 0.00 ^a^	0.969
	BPC_19_	2.65 ± 0.08 ^a^	0.08 ± 0.01 ^a^	0.60 ± 0.02 ^a^	0.970
10	BPC_0_	2.95 ± 0.14 ^a^	0.70 ± 0.22 ^a^	0.96 ± 0.03 ^a^	0.973
	BPC_2_	2.89 ± 0.03 ^a^	0.45 ± 0.04 ^a^	0.92 ± 0.01 ^a^	0.977
	BPC_6_	2.79 ± 0.03 ^a^	0.46 ± 0.04 ^a^	0.93 ± 0.01 ^a^	0.976
	BPC_9_	2.84 ± 0.21 ^a^	0.61 ± 0.09 ^a^	0.95 ± 0.01 ^a^	0.975
	BPC_13_	3.09 ± 0.30 ^a^	0.50 ± 0.18 ^a^	0.93 ± 0.04 ^a^	0.967
	BPC_19_	3.03 ± 0.21 ^a^	0.55 ± 0.17 ^a^	0.94 ± 0.03 ^a^	0.967
15	BPC_0_	2.86 ± 0.16 ^a^	1.32 ± 0.27 ^a^	1.02 ± 0.01 ^a^	0.981
	BPC_2_	2.75 ± 0.05 ^a^	1.29 ± 0.21 ^a^	1.02 ± 0.01 ^a^	0.974
	BPC_6_	2.75 ± 0.05 ^a^	1.22 ± 0.10 ^a^	1.01 ± 0.01 ^a^	0.975
	BPC_9_	2.91 ± 0.27 ^a^	1.30 ± 0.34 ^a^	1.02 ± 0.02 ^a^	0.973
	BPC_13_	2.88 ± 0.04 ^a^	1.75 ± 0.45 ^a^	1.04 ± 0.02 ^a^	0.976
	BPC_19_	2.83 ± 0.18 ^a^	1.32 ± 0.25 ^a^	1.02 ± 0.01 ^a^	0.975
20	BPC_0_	2.72 ± 0.17 ^a^	3.15 ± 0.57 ^a^	1.06 ± 0.01 ^a^	0.980
	BPC_2_	2.80 ± 0.11 ^a^	2.38 ± 0.22 ^a^	1.04 ± 0.00 ^a^	0.978
	BPC_6_	2.90 ± 0.11 ^a^	2.44 ± 0.34 ^a^	1.05 ± 0.01 ^a^	0.977
	BPC_9_	2.85 ± 0.01 ^a^	3.31 ± 0.94 ^a^	1.06 ± 0.02 ^a^	0.979
	BPC_13_	2.70 ± 0.04 ^a^	3.00 ± 0.07 ^a^	1.06 ± 0.00 ^a^	0.976
	BPC_19_	2.83 ± 0.11 ^a^	2.28 ± 0.34 ^a^	1.04 ± 0.01 ^a^	0.969
25	BPC_0_	2.83 ± 0.05 ^a^	5.29 ± 0.31 ^a^	1.07 ± 0.00 ^a^	0.985
	BPC_2_	2.85 ± 0.13 ^a^	4.61 ± 0.62 ^a^	1.06 ± 0.01 ^a^	0.981
	BPC_6_	2.98 ± 0.13 ^a^	4.78 ± 0.62 ^a^	1.06 ± 0.01 ^a^	0.982
	BPC_9_	2.73 ± 0.12 ^a^	6.60 ± 0.56 ^a^	1.08 ± 0.01 ^a^	0.986
	BPC_13_	2.90 ± 0.08 ^a^	6.13 ± 0.38 ^a^	1.08 ± 0.00 ^a^	0.981
	BPC_19_	2.80 ± 0.11 ^a^	5.65 ± 0.51 ^a^	1.07 ± 0.00 ^a^	0.979

Values are the mean ± SD (*n* = 3). Different superscript letters within a given cooling rate indicate a significant difference (*p* < 0.05).

**Table 4:** Parameters calculated at various relative crystallinities (*X*) based on the Avrami–Ozawa model.

*X* (%)	BPCs	*F*(*T*)	*α*	*R* ^2^
20	BPC_0_	5.7 ± 0.3 ^b^	1.21 ± 0.12 ^a^	0.977
	BPC_2_	6.3 ± 0.3 ^ab^	1.15 ± 0.02 ^a^	0.992
	BPC_6_	6.2 ± 0.1 ^ab^	1.18 ± 0.03 ^a^	0.994
	BPC_9_	5.8 ± 0.2 ^ab^	1.16 ± 0.09 ^a^	0.993
	BPC_13_	5.8 ± 0.3 ^ab^	1.16 ± 0.05 ^a^	0.993
	BPC_19_	6.6 ± 0.5 ^a^	1.06 ± 0.05 ^a^	0.991
40	BPC_0_	7.3 ± 0.3 ^a^	1.27 ± 0.07 ^a^	0.988
	BPC_2_	8.0 ± 0.3 ^a^	1.18 ± 0.01 ^ab^	0.993
	BPC_6_	7.9 ± 0.2 ^a^	1.21 ± 0.04 ^ab^	0.995
	BPC_9_	7.3 ± 0.3 ^a^	1.19 ± 0.08 ^ab^	0.994
	BPC_13_	7.2 ± 0.3 ^a^	1.19 ± 0.05 ^ab^	0.994
	BPC_19_	8.0 ± 0.5 ^a^	1.08 ± 0.05 ^b^	0.993
60	BPC_0_	8.8 ± 0.2 ^a^	1.31 ± 0.08 ^a^	0.989
	BPC_2_	9.5 ± 0.3 ^a^	1.21 ± 0.01 ^ab^	0.993
	BPC_6_	9.6 ± 0.3 ^a^	1.23 ± 0.04 ^ab^	0.996
	BPC_9_	8.7 ± 0.4 ^a^	1.21 ± 0.08 ^ab^	0.994
	BPC_13_	8.6 ± 0.3 ^a^	1.23 ± 0.04 ^ab^	0.995
	BPC_19_	9.3 ± 0.5 ^a^	1.11 ± 0.05 ^b^	0.995
80	BPC_0_	11.0 ± 0.2 ^a^	1.35 ± 0.07 ^a^	0.990
	BPC_2_	11.5 ± 0.3 ^a^	1.24 ± 0.01 ^ab^	0.992
	BPC_6_	11.7 ± 0.5 ^a^	1.25 ± 0.04 ^ab^	0.996
	BPC_9_	10.5 ± 0.6 ^a^	1.25 ± 0.07 ^ab^	0.995
	BPC_13_	10.4 ± 0.3 ^a^	1.27 ± 0.05 ^ab^	0.994
	BPC_19_	10.9 ± 0.6 ^a^	1.14 ± 0.05 ^b^	0.995

Values are the mean ± SD (*n* = 3). Different superscript letters (a and b) within a given *X* indicate a significant difference (*p* < 0.05).
